# Effects of testosterone and metformin on the GlycanAge index of biological age and the composition of the IgG glycome

**DOI:** 10.1007/s11357-024-01349-z

**Published:** 2024-10-04

**Authors:** Martina Vinicki, Tea Pribić, Frano Vučković, Azra Frkatović-Hodžić, Isaac Plaza-Andrades, Francisco Tinahones, Joseph Raffaele, José Carlos Fernández-García, Gordan Lauc

**Affiliations:** 1 Glycoscience Research Laboratory, Genos Ltd, Borongajska Cesta 83H, Zagreb, Croatia; 2 Grupo de Oncología Traslacional, Centro de Investigación Médico-Sanitario (CIMES), Laboratorio Inmunobiota, Malaga, Spain; 3 Department of Endocrinology and Nutrition, Faculty of Medicine, Hospital Virgen de La Victoria (IBIMA), University of Malaga, Malaga, Spain; 4 Spanish Biomedical Research Center in Physiopathology of Obesity and Nutrition (CIBERObn), Instituto de Salud Carlos III, Madrid, Spain; 5 PhysioAge Systems, New York, NY 10019 USA; 6 Raffaele Medical, New York, NY 10019 USA; 7 Department of Endocrinology and Nutrition, Faculty of Medicine, Hospital Regional Universitario de Malaga (IBIMA), University of Malaga, Malaga, Spain; 8 Faculty of Pharmacy and Biochemistry, University of Zagreb, Ante Kovačića 1, Zagreb, Croatia

**Keywords:** IgG, Glycosylation, Testosterone, Metformin, Aging

## Abstract

With aging, the body’s ability to maintain regular functions declines, increasing susceptibility to age-related diseases. Therapeutic interventions targeting the underlying biological changes of aging hold promise for preventing or delaying multiple age-related diseases. Metformin, a drug commonly used for diabetes treatment, has emerged as a potential gerotherapeutic agent due to its established safety record and preclinical and clinical data on its anti-aging effects. Glycosylation, one of the most common and complex co- and post-translational protein modifications, plays a crucial role in regulating protein function and has been linked to aging and various diseases. Changes in immunoglobulin G (IgG) glycosylation patterns have been observed with age, and these alterations may serve as valuable biomarkers for disease predisposition, diagnosis, treatment monitoring, and overall health assessment. In this study, we analyzed the IgG glycosylation patterns of white men from Europe, aged 29–45 years, under treatment with metformin, testosterone, metformin plus testosterone, and placebo (trial registration number NCT02514629, 2013/07/04), and investigated the longitudinal changes in glycosylation over time. We observed statistically significant differences in the IgG glycome composition between participants on testosterone therapy and placebo, with decreased agalactosylation and increased galactosylation and sialylation. However, metformin therapy did not result in statistically significant changes in glycosylation patterns. These findings contribute to our understanding of the impact of therapeutic interventions on IgG glycosylation and confirm the value of IgG glycosylation as a significant biomarker, capable of assessing biological age using the GlycanAge index and providing insight into overall health compared to chronological age.

**Supplementary Information:**

The online version contains supplementary material available at 10.1007/s11357-024-01349-z.

## Introduction

The aging process is characterized by a gradual decline in the body’s ability to maintain normal functioning, resulting in an increased susceptibility to various diseases including cancer, diabetes, and cardiovascular and neurodegenerative disorders [1]. Therapeutic interventions targeting the underlying biological changes of aging can potentially prevent or delay multiple age-related diseases with a single treatment [2]. While significant progress has been made in understanding the genetic pathways and biochemical processes linked to aging, one of the critical challenges in aging research is deciphering the interconnectedness of the various hallmarks of aging, such as genetic and epigenetic modifications, and their individual contributions to the aging process [1].

Gerotherapeutic drugs have emerged as promising candidates for targeting the aging process. In this line, metformin, a medication commonly used to treat type 2 diabetes, has gained recognition as an antiaging agent due to abundant preclinical data and human clinical studies spanning over 60 years [2, 3]. In accordance with this, metformin is among the leading gerotherapeutic agents currently being tested in human clinical trials to assess its impact on the aging process [2]. Initially considered a caloric restriction mimetic [4], metformin has demonstrated the potential to extend lifespan and reduce the occurrence of age-related diseases [3, 5, 6].

Similar to the aging process, glycosylation is influenced by a complex interplay of both genetic and environmental factors [7]. Glycosylation is one of the most common and complex co- and post-translational protein modifications, playing a crucial role in the regulation of glycoprotein biological activities [8]. IgG, a well-studied protein, has been identified as an ideal model for investigating glycosylation in aging research [9]. IgG glycosylation is a tightly regulated process, influenced by both the central and paracentral dogmas through glycogenes [10], and any disruptions of this process can lead to changes in glycosylation patterns that have been observed in aging as well as in various diseases [11] that can be caused by obesity, among others. Being a multifactorial chronic disease, obesity has evolved into a major global public health issue [12]. According to the World Health Organization (WHO), obesity is defined as abnormal or excessive fat accumulation that poses a health risk, identified by a body mass index (BMI) exceeding 30. When the organism’s homeostasis is disrupted, either due to lifestyle changes or pathophysiological processes, it enters a state of inflammation. Inflammaging is a persistent, low-grade inflammatory state associated with aging in which glycosylation changes occur, often resembling those seen in inflammatory disorders, likely resulting from the immune system’s ongoing exposure to inflammatory triggers of both internal and external origin [13]. Therefore, IgG glycosylation may serve as a valuable tool for enhancing the accuracy of current biomarkers for disease predisposition, diagnosis, treatment monitoring, and prognosis, as well as assessing a person’s overall health state, and biological age [11].

Studies investigating changes in the IgG N-glycome over time have validated the observed alterations in IgG galactosylation patterns that occur with age [14]. Pučić et al. showed a reduction in galactosylation and sialylation and an increase in bisecting N-Acetylglucosamine (GlcNAc) with advancing age [15]. Several epidemiological studies have shown metformin’s gerotherapeutic effect in reducing the occurrence of age-related diseases and overall mortality [5, 6]. Also, association studies indicate that metformin may lower the risk of several age-related conditions [3]. A decrease in sialylated structures and an increase in agalactosylated structures may be associated with a decrease in terminally galactosylated structure, which indicates that the majority of IgG molecules remain in the G0 glycosylation state and therefore could be interpreted as an IgG glycoprofile with high inflammatory potential [11]. Combining these glycan features can provide better predictive value [16] than individual markers of biological aging. The findings by Krištić et al. suggest that employing IgG glycosylation patterns to evaluate the GlycanAge index of biological age might function as a holistic indicator reflecting an individual’s overall health status when contrasted with their chronological age [14]. It is important to note that within the concept of inflammation, IgG glycans are not only considered biomarkers but also one of the molecular effectors of the aging process [17].

Considering previous research and the importance of glycosylation in aging, we performed an IgG N-glycome analysis in a randomized clinical trial including men with obesity and low levels of testosterone that were treated with metformin, testosterone, or both. The primary objective of our study was to investigate the differences in IgG N-glycosylation occurring during 12 months of treatment. Additionally, we evaluated changes in the IgG N-glycome in a cross-sectional study that included men treated with metformin, testosterone, the combination of both, or no treatment, to verify replication of the results obtained in the clinical trial.

## Study population and methods

### Participants

The randomized clinical trial consisted of 82 male participants, predominantly white men from Europe, aged 29–45 years, with obesity (BMI ≥ 30 kg/m^2^) and low testosterone levels (total testosterone below 230 ng/dl (8 nmol/l) or free testosterone below 70 pg/ml (240 pmol/l)). Participants with diabetes mellitus (identified by fasting glucose ≥ 126 mg/dl (7 mmol/l), glycated hemoglobin ≥ 6.5% or use of anti-diabetic medication)), malignancy, cardiovascular disease, or other significant health conditions were excluded from the study [18]. These patients were randomized into four groups, i.e., metformin (*n* = 19), testosterone (*n* = 24), metformin plus testosterone (*n* = 20), or placebo (*n* = 19), and were followed for 12 months. Plasma samples, three per participant, were collected at three different time points (basal, 6 months, and 12 months), with collections conducted at the Department of Endocrinology and Nutrition at the Virgen de la Victoria University Hospital (Malaga, Spain). The study protocol was approved by the institutional review boards and the local ethics committee, registered with ClinicalTrials.gov (NCT02514629).

On the other hand, 129 plasma samples in the cross-sectional general population study were collected at a single time point, one sample per participant, at Raffaele Medical (New York, USA), chronological age mean = 59 and SD = 13, to verify the replication of the results obtained in the clinical trial. Participants were grouped based on the therapy they were receiving: testosterone (*n* = 66), metformin (*n* = 5), both testosterone and metformin (*n* = 12), and no therapy (*n* = 46).

Before glycan analysis, participant info has been anonymized by introducing a unique participant ID which was used as an identifier during laboratory glycan analysis. All participants signed informed consent forms, and ethical committees of both institutions approved the research. The study was conducted in accordance with the Declaration of Helsinki.

## Methods

### Isolation of IgG from human plasma

Samples were randomly distributed across three 96-well plates, with each plate containing samples, six standards, and one blank. To isolate IgG from a 25 μL of plasma sample, the *CIM® r-Protein G LLD 0.05 mL Monolithic 96-well Plate (2 µm channels)* (BIA Separations, Slovenia, Cat No. 120.1012–2) was used following the protocols initially described previously [15, 19], modified to one-quarter of the solutions specified in the original protocol. IgG was eluted from a monolithic plate with 0.1 M formic acid, pH 2.5, and the eluates were collected in a 96-deep-well plate and neutralized with 1 M ammonium bicarbonate (Acros Organic, USA). The vacuum-assisted setup utilized a manual system consisting of a multichannel pipette, a vacuum manifold (Pall Corporation, USA), and a vacuum pump (Pall Corporation, USA), with pressure reductions of approximately 5 inHg during sample application and IgG elution. Following the isolation of IgG, the monolithic plate was stored in 20% (v/v) EtOH in 20 mM TRIS + 0.1 M NaCl, pH 7.4 at 4 °C. Subsequently, 20 μL of IgG was aliquoted in a PCR plate (Thermo Scientific, UK) and dried in a vacuum centrifuge. For the purpose of IgG N-glycan analysis by capillary gel electrophoresis with laser-induced fluorescence (CGE-LIF), deglycosylation, released N-glycan labeling, and clean-up of labeled IgG N-glycans were performed using modified protocols described elsewhere [20, 21].

#### Glycan release, labeling, and clean-up

Isolated IgG samples were dried in a vacuum concentrator, diluted with 3 μL of 1.66 × PBS (w/v), and denatured in 4 μL 0.5% SDS (w/v) (Sigma-Aldrich, USA) by incubation at 65 °C for 10 min. Following incubation, 2 μL of 4% Igepal-CA630 (Sigma-Aldrich, USA) was added to the samples and incubated on a shaker for approximately 5 min. For glycan release, 1 μL of the enzyme mixture (1.2U of PNGase F (Promega, USA) in 1 μL 5 × PBS) per sample was added and incubated for 3 h at 37 °C. After incubation, the IgG deglycosylation mix was dried in a vacuum concentrator for 1 h, diluted with 2 μL of ultra-pure water, and left on a shaker for approximately 5 min to prepare the labeling mixture for labeling the released N-glycans. The labeling mixture was freshly prepared by combining 2 μL of 30 mM 8-aminopyrene-1,3,6-trisulfonic acid trisodium salt (APTS) (Synchem, DE) in 3.6 M citric acid (Sigma-Aldrich, USA) with the 2 μL of 1,2 M 2-picoline borane in dimethyl sulfoxide (DMSO) (Sigma-Aldrich, USA) per sample. Mixing was achieved by vortexing several times, followed by 16 h incubation at 37 °C. The labeling reaction was stopped by adding 100 μL of cold 80% acetonitrile (Carlo Erba, Spain) prior to the clean-up procedure by hydrophilic interaction chromatography based solid phase extraction (HILIC-SPE).

For the clean-up procedure, 200 μL of Bio-Gel P-10 slurry was added on a 0.2 μm wwPTFE AcroPrep filter plate (Pall Corporation, USA) per well and used as the stationary phase. The wells containing Biogel P-10 were prewashed with 200 μL ultrapure water and 200 μL 80% cold ACN (v/v) (Carlo Erba, Spain), three times for each step. The samples were loaded into the wells, and after a 5-min incubation on a shaker, samples were washed with 5 × 200 μl 80% ACN containing 100 mM TEA, pH 8,5 (Carlo Erba, Spain/MilliporeSigma, USA) followed by 3 × 200 μl 80% ACN (Carlo Erba, Spain) after 2 min of incubation at room temperature. IgG N-glycans were eluted with a total of 500 μl of ultrapure water after 5-min incubation at room temperature after each step, and combined eluate fractions were stored at – 20 °C until use.

#### CGE-LIF analysis and data processing

The IgG N-glycoprofiling by the CGE-LIF method was performed using an Applied Biosystems 3500 Genetic Analyser (Thermo-Fischer Scientific, USA) equipped with 50-cm-long 8-capillary array (Thermo-Fischer Scientific, USA). Polymer POP-7 (Thermo-Fischer Scientific, USA) was used as a separation matrix in capillaries. In a 96-well MicroAmp Optical 96-Well reaction plate, 2 μl of APTS-labeled N-glycans were combined with 8 μl of HiDi formamide, ensuring a thorough and uniform resuspension. The instrumental method was created by setting the operating parameters as follows: injection time, 9 s; injection voltage, 15 kV; run voltage, 19,5 kV; oven temperature, 60 °C; and working time, 1000 s. The obtained electropherograms were integrated in the same manner into 27 peaks using Waters Empower 3 software. The composition of each peak has been previously determined (Supplement 1) [22].

### Statistical analysis

Normalization and batch correction were performed on CGE glycan data to eliminate experimental variation in measurements. To remove experimental noise and make the glycan peak measurements comparable across samples regardless of their absolute intensities, a total area normalization was performed. The peak area of each of the 27 glycan structures obtained directly was divided by the total area of the corresponding electropherogram and multiplied by 100, with each peak being expressed as a percentage of the total integrated area. Before batch correction, normalized glycan measurements were log-transformed due to the right skewness of their distributions and the multiplicative nature of batch effects. A batch correction was performed on logarithmically transformed measurements using the ComBat method (R package sva), where the technical source of variation, the number of sample plates, was modeled as a batch covariate. This was performed for each glycan peak. Estimated batch effects were subtracted from logarithmically transformed measurements to provide measurement correction for experimental noise.

Longitudinal analysis of samples through their observation period was performed by implementing a linear mixed-effects model where time was modeled both as a fixed effect and random slope, the interaction between time and therapy was modeled as a fixed effect, and individual sample ID was modeled as a random intercept. Prior to the analyses, glycan variables were all transformed to a standard normal distribution by the inverse transformation of ranks to normality (R package “GenABEL”; function rntransform). Using rank-transformed variables makes the estimated effects of different glycans comparable as these will have the same standardized variance. In the cross-sectional study, the differences between controls and individuals receiving the therapy were tested using a linear regression model with glycan traits as dependent variables and metformin status as an independent variable while controlling for testosterone status and vice versa. Chronological age was included as an additional covariate. The Benjamini–Hochberg procedure was used to control the false discovery rate (FDR) at the specified level of 0.05. Data were analyzed and visualized using the R programming language (version 3.5.2). GlycanAge was calculated in cross-sectional study by performing lasso regression for feature selection among most abundant IgG glycans using *glmnet()* function in R package “glmnet.” Samples used for training of the model included male samples coming from the Raffaele Medical clinic with a total sample size of *N* = 198. Following glycans were selected and used in GlycanAge calculation: FA2, FA2G1, FA2BG1, FA2G2, and FA2BG2S2. Association of GlycanAge with metformin and testosterone treatment was tested using linear regression while controlling for chronological age.

## Results

IgG N-glycome composition in the clinical trial was analyzed in 82 participants during 1 year of treatment, comprising 19 men on metformin therapy, 24 men on testosterone therapy, 20 men on combination therapy (metformin plus testosterone), and 19 men on placebo. The cross-sectional study, which was used as a proxy for validation of the findings, consisted of 5 participants on metformin, 66 participants on testosterone, 12 on both therapies, and 46 on no therapy. All participants were male, and a basic description of the cohorts investigated are provided in Table 1 and Table 2.

**Table 1:** Descriptive information about participants included in the clinical trial

	Clinical trial			
	Testosterone group	Metformin group	Metformin + testosterone group	Placebo group
*n*	24	19	20	19
Age	41^1^ (36–45)	38^1^ (29–42)	38^1^ (34–43)	40^1^ (34–43)
HbA1c	5.53^2^ (0.30)	5.38^2^ (0.39)	5.44^2^ (0.34)	5.64^2^ (0.38)

^1^Median (IQR), ^2^mean (SD)

**Table 2:** Descriptive information about participants included in the cross-sectional study

	Cross-sectional study			
	Testosterone group	Metformin group	Metformin + testosterone group	No treatment group
*n*	66	5	12	46
Age	61^1^ (56–66)	67^1^ (66–72)	70^1^ (67–76)	50^1^ (40–63)
HbA1c	5.37^2^ (0.36)	5.58^2^ (0.27)	6.48^2^ (1.24)	5.40^2^ (0.33)

^1^Median (IQR), ^2^mean (SD)

**Fig. 1:**
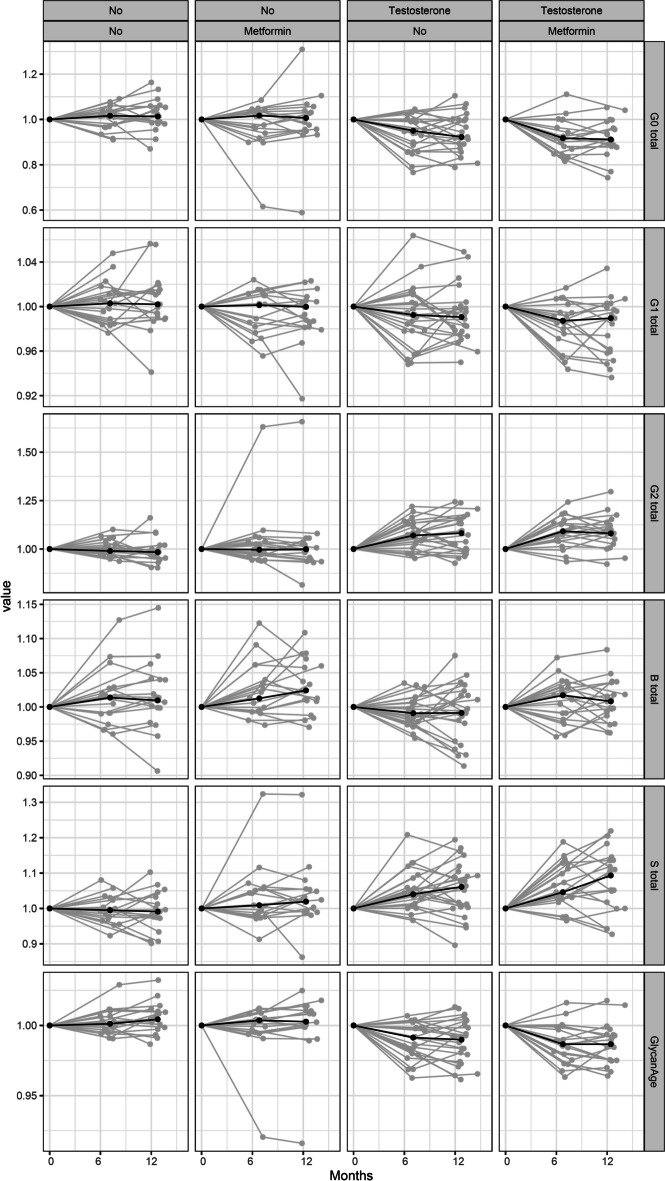
Alterations in IgG glycome composition and GlycanAge index of biological age in participants on placebo (*n* = 19), testosterone (*n* = 24), metformin (*n* = 19), or testosterone + metformin (*n* = 20) therapy followed for 12 months in the clinical trial. Standardized glycan measurements are represented on the *y*-axis, while time in months is presented on the *x*-axis. Black dots represent 6 months, cohort-specific averages of standardized glycan measurements. Statistical tests used: normalization and batch correction, a linear mixed-effects model. G0, agalactosylated N-glycans; G2, digalactosylated N-glycans; S, sialylated N-glycans; B, bisecting GlcNAc N-glycans. Additional information is available in Table 3

Analyzing clinical trial samples, we found a consistent decrease in the level of glycans without galactose (G0) in those men treated with testosterone (effect =  − 0.0302; *p* = 0.0015), accompanied by an increase in digalactosylation (G2) (effect = 0.0337; *p* = 0.0014) and sialylation (S) (effect = 0.0261; *p* = 0.0015). Participants on testosterone therapy exhibited a statistically significant decrease in their biological age when assessed using the GlycanAge index of biological age (effect =  − 0.032, *p* = 0.0008). On the other hand, during the course on metformin therapy, we did not observe statistically significant changes in agalactosylation (G0) (effect =  − 0.0087; *p* = 0.3215), or digalactosylation (G2) (effect = 0.0092; *p* = 0.3215). The level of sialylation (S) increased (effect = 0.0123; *p* = 0.1367) (Table 3,Fig. 1).

**Table 3:** Statistical analysis of IgG glycome composition changes and GlycanAge index of biological age during the course of testosterone (*n* = 24) and metformin (*n* = 19) therapy in the clinical trial. Statistical tests used: normalization and batch correction, a linear mixed-effects model, where effect model were time

Clinical trial								
	Testosterone therapy				Metformin therapy			
Glycan	Effect	SE	pval	p.adj	Effect	SE	pval	p.adj
G0 total	− 0.0302	0.0082	0.0005	0.0015	− 0.0087	0.0083	0.2947	0.3215
G2 total	0.0337	0.0087	0.0002	0.0014	0.0092	0.0087	0.2914	0.3215
B total	− 0.0083	0.0044	0.0617	0.1058	0.0062	0.0044	0.1628	0.2171
S total	0.0261	0.0072	0.0005	0.0015	0.0123	0.0072	0.0911	0.1367
GlycanAge	− 0.032	0.0076	0.0001	0.0008	− 0.0067	0.0076	0.3772	0.3772

**Fig. 2:**
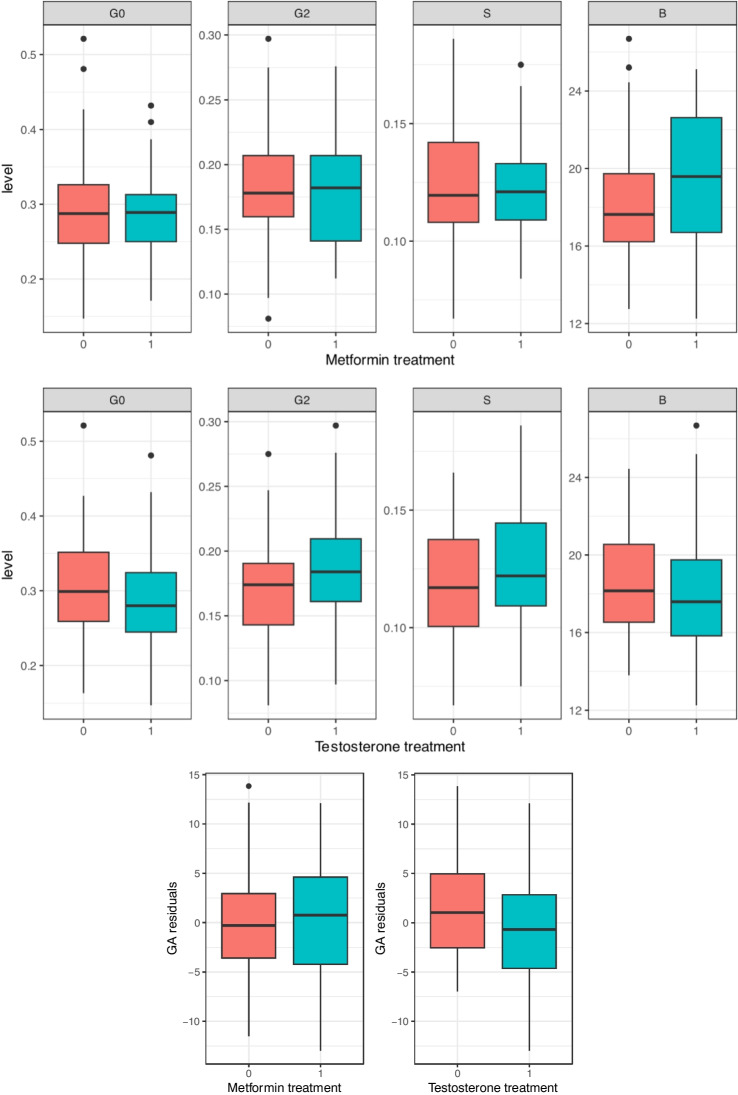
Differences in IgG glycome composition, and GlycanAge index of biological age, in participants on testosterone (*n* = 66), metformin (*n* = 5), and combined (*n* = 12) therapies in comparison with participants not receiving the therapy (*n* = 46) in the cross-sectional study. Groups are represented on X axis; residuals of GlycanAge index of biological age corrected for chronological age are represented. Data are shown as boxplots. The boxes represent the 25th to 75th percentiles in the glycan data. The line inside the box is the median value. Lines outside the box represent the 10th to 90.^th^ percentile. Black dots represent outliers. G0, agalactosylated N-glycans; G2, digalactosylated N-glycans; S, sialylated N-glycans; B, bisecting GlcNAc N-glycans. Additional information is available in Table 4

The analysis of differences between participants on metformin therapy and participants without treatment, and participants on testosterone therapy and controls during the cross-sectional study, was performed using a linear regression model while controlling for testosterone and metformin status, respectively. A statistically significant difference in the IgG glycome composition of participants on testosterone therapy was observed (Table 4,Fig. 2). Lower levels of agalactosylation (G0) were observed in participants on testosterone therapy (beta =  − 0.6508; adjusted *p* = 0.0013), while the level of digalactosylation (G2) was higher (beta = 0.6472; *p* = 0.0013). Additionally, the levels of sialylated (S) IgG glycan structures in participants on testosterone therapy were higher (beta = 0.7024; *p* = 0.0013), while biological age of participants on testosterone therapy was lower as determined by the GlycanAge index of biological age (beta =  − 0.3602, *p* = 0.0394). No statistically significant effects of metformin therapy were observed.

**Table 4:** Statistical analysis of IgG glycome composition changes and GlycanAge index of biological age during testosterone (*n* = 66) and metformin (*n* = 5) therapy in the cross-sectional study. Statistical tests used: lasso regression; linear regression, where beta is treatment

Cross-sectional study								
	Testosterone therapy				Metformin therapy			
Glycan	Beta	SE	pval	p.adj	Beta	SE	pval	p.adj
G0 total	− 0.6508	0.1755	0.0003	0.0013	− 0.3292	0.2483	0.1874	0.3123
G2 total	0.6472	0.1776	0.0004	0.0013	0.2807	0.2513	0.2662	0.3609
B total	− 0.5148	0.183	0.0057	0.0142	0.0887	0.259	0.7326	0.814
S total	0.7024	0.178	0.0001	0.0013	0.2684	0.2519	0.2887	0.3609
GlycanAge	− 0.3602	0.1524	0.0197	0.0394	− 0.0432	0.2157	0.8417	0.8417

## Discussion

The alternations in the IgG glycosylation pattern associated with aging have been well-documented. However, the underlying mechanisms behind these changes and whether they are a consequence of other age-related processes or play a functional role in the aging process are not fully understood [23]. The glycosylation process, influenced by both the central and paracentral dogmas through glycogenes, is crucially organized [10]. Disruptions to this process, whether through genetic lesions like mutations, translocations, chromosomal changes, telomere shortening, or epigenetic alterations in DNA and histones, significantly contribute to the aging process [1]. Glycans play a significant role in the IgG molecule, influencing its properties based on their presence, absence, and composition.

In this study, we analyzed IgG glycosylation in plasma samples from men with obesity and low testosterone levels that participated in a double-blind, placebo-controlled clinical trial. These participants were randomized to treatment with testosterone, metformin, metformin plus testosterone or placebo for 52 weeks. Additionally, we validated our results by conducting a cross-sectional analysis using samples from another cohort, which underwent the same treatment therapies.

Our main findings indicate consistent differences in the IgG glycome composition between participants on treatment with testosterone and metformin therapy, both in the clinical trial and in the cross-sectional study. Hence, participants on testosterone therapy in the clinical trial exhibited a consistent decrease in the level of agalactosylated glycans, while digalactosylation and sialylation were increased. However, agalactosylation and digalactosylated, as well as sialylation, did not statistically significantly change during the metformin therapy. The cross-sectional study also demonstrated lower levels of agalactosylated glycans and higher digalactosylation and sialylation in participants under testosterone therapy. However, no statistically significant differences were observed in agalactosylation, digalactosylated N-glycans, or sialylation in participants treated with metformin therapy in the cross-sectional study. The results obtained in the clinical trial were confirmed by the findings from the cross-sectional study.

Numerous studies have reported similar changes in agalactosylated and digalactosylated IgG glycans with aging [14, 24–26]. Yamada et al. found increased levels of agalactosylated glycans with age in both genders [24], which is in correlation with the study by Pučić Baković and colleagues [25]. They also reported a decrease in the relative abundance of digalactosylated glycans with age [25]. Krištić et al. conducted a large-scale study on European populations and observed that the relative abundances of agalactosylated glycans increased with age, while digalactosylated glycans decreased with age [14]. Furthermore, the study by Yu et al. reported an increase in levels of agalactosylated and a decrease in levels of digalactosylated glycans with age [26]. Age-related alterations in sialylated glycans were observed. A large-scale study by Pučić Baković et al. has reported a negative correlation between sialylated glycan levels and age [25]. Additionally, Krištić et al. reported a decrease in the most prevalent sialylated IgG glycan and its desialylated counterpart with aging [14]. These consistent findings across different populations suggest that age-related changes in sialylation may play a role in the aging process [14, 17, 25, 27], showing significant sex dependence [28].

Several studies have extensively investigated changes in glycans that contain bisecting GlcNAc with age. These studies revealed significant variation in the pattern of change of bisecting GlcNAc IgG glycans as people grow older [17, 25, 27, 29, 30]. Ruhaak et al. reported that the ratio of bisecting to non-bisecting digalactosylated glycans tended to increase with age [29]. Additionally, two large-scale studies, one by Chen et al., in a Chinese population [30], and another by Pučić Baković et al., in a European population [25], reported that the level of bisecting glycans increased with age.

In our clinical trial, we closely examined individuals undergoing testosterone therapy and those undergoing metformin therapy. We observed a decrease in bisecting glycans in individuals undergoing testosterone therapy, while those on metformin therapy showed an increase in bisecting IgG glycans. However, it is important to note that these observed changes were not statistically significant in any of the applied therapies in the clinical trial. In the cross-sectional study, the level of N-glycans with bisecting GlcNAc was indeed lower in individuals receiving testosterone therapy. Furthermore, the relationship between core fucosylated IgG glycans and age yielded inconsistent results. While some studies reported no or weak correlation between core-fucosylated glycans and age in adults, the majority of studies did not identify significant associations [17, 25, 27, 29, 30].

This comprehensive study sheds light on the effects of testosterone and metformin therapy on IgG N-glycome composition and reveals consistent differences in glycosylation patterns between the two therapy groups. Participants undergoing testosterone therapy displayed a decrease in agalactosylated glycans and an increase in digalactosylated glycans, accompanied by increased sialylation. However, metformin therapy did not significantly alter galactosylation or sialylation. These findings align with previous studies on age-related changes in IgG glycosylation, suggesting a potential connection between therapy-induced glycosylation patterns and the aging process. In line with this, our work highlights the intricate interplay among therapy, aging, and glycan-mediated molecular mechanisms. Considering this study’s exclusive focus on male participants, it would be intriguing to investigate the effects of these therapies on females. This is particularly relevant as modification of IgG N-glycosylation is recognized as one of the mechanisms through which sex hormones influence the immune system, so it is important to consider hormonal changes when examining these effects in women [31].

Our study stands out due to its significant strengths, primarily the large sample size and the longitudinal nature of our data. To the best of our knowledge, this is the first study to investigate longitudinal changes in total IgG glycome analysis in a cohort of men with obesity and low levels of testosterone, treated with metformin, testosterone, or both. The study’s robustness is underscored by its adherence to the gold standard in clinical research—a randomized, double-blind, placebo-controlled design—which decreases the risk for bias or confounding factors. Additionally, the judicious selection of nondiabetic individuals with obesity and no chronic diseases or cardiovascular issues enhances the study’s internal validity. Furthermore, the 1-year duration of the clinical trial provides a comprehensive view of the long-term effectiveness of the tested therapies, a crucial element in understanding their impact. Moreover, the utilization of high-throughput analysis in sample processing adds a further layer of robustness to the research. Nonetheless, it is essential to acknowledge the study’s limitations. Our findings are based on three sampling points during 12 months in the clinical study and one point during the cross-sectional study. Ideally, both cohorts should include the same number of sampling points over the same time period. Although we have used the cross-sectional study primarily as a validation of findings from the longitudinal study, the small sample size may be of concern due to its implications for statistical power and reliability. Therefore, a larger sample size study is warranted to further confirm these findings.

Overall, our findings underscore the role of the IgG glycome as both a biomarker and a functional contributor to aging and age-related disease. A study conducted by Krištić et al. in 2014 [14] demonstrated that the IgG glycosylation pattern could be used to estimate a person’s biological age with a predictor error of ± 9.7 years, explaining nearly 60% of the variation in chronological age. These findings suggest that using IgG glycosylation patterns to estimate the GlycanAge index of biological age could provide an overall indicator of an individual’s health status when compared to their chronological age. In line with this, one group developed an index for the prediction of cardiovascular risk that shows an even greater development of glycomedicine [16]. Additionally, some findings indicate that people in developing countries may experience an increased onset of inflammatory conditions and accelerated biological aging due to exposure to the environmental factors [32]. However, further research is necessary to unveil the functional significance of these glycosylation changes and explore the broader implications of IgG glycosylation as a biomarker and effector of the aging process. Understanding the impact of therapeutic interventions on IgG glycosylation may contribute to the development of personalized approaches for disease prevention, diagnosis, and treatment based on individualized glycoprofiles.

## Supplementary Information

Below is the link to the electronic supplementary material.

Supplementary file1 (DOCX 1032 KB)

## Abbreviations

*ACN*: Acetonitrile
*APTS*: 8-Aminopyrene-1,3,6-trisulfonic acid trisodium salt
*BMI*: Body mass index
*CGE-LIF*: Capillary gel electrophoresis with laser-induced fluorescence
*DMSO*: Dimethyl sulfoxide
*DNA*: Deoxyribonucleic acid
*Fab*: Fragment antigen-binding
*Fc*: Fragment crystallizable
*GlcNAc*: N-Acetylglucosamine
*HiDi*: Formamide
*HILIC-SPE*: Hydrophilic interaction liquid chromatography solid-phase extraction
*IgG*: Immunoglobulin G
*PBS*: Phosphate buffered saline
*PNGase F*: Peptide N-glycosidase F
*SDS*: Sodium dodecyl sulfate
*TEA*: Triethylamine
*WHO*: World Health Organization
