# Supplementary material for: Effects of testosterone and metformin on the GlycanAge index of biological age and the composition of the IgG glycome
Source: GeroScience. 2024 Oct 4;47(2):1777–88. doi: 10.1007/s11357-024-01349-z (PMC11979073; doi:10.1007/s11357-024-01349-z)
Supplement: Supplementary file 1 — Supplementary file1 (DOCX 1032 KB) [file 11357_2024_1349_MOESM1_ESM.docx]

**Supplementary Table 1. Formulas for calculation of derivated traits.**

| **Derived trait** | **Formula** |
| --- | --- |
| G0 | P14+P15+P18 |
| G1 | P16+P17+P19+P20+P21+P22+P23+P24 |
| G2 | P25+P26+P27 |
| S | P1+P2+P3+P4+P5+P6+P7+P8+P9+P10+P11+P12+P13 |
| B | P2+P4+P11+P13+P14+P18+P19+P20+P23+P24+P25+P27 |

**Supplementary Table 2. Glycan structures under 27 CGE peaks.**

Structure abbreviations: all N-glycans have core sugar sequence consisting of two N-acetylglucosamines (GlcNAc) and three mannose residues; F indicates a core fucose α1–6 linked to the inner GlcNAc; Mx, number (x) of mannose on core GlcNAcs; Ax, number of antenna (GlcNAc) on trimannosyl core; A2, biantennary glycan with both GlcNAcs as β1–2 linked; B, bisecting GlcNAc linked β1–4 to β1–3 mannose; Gx, number of β1–4 linked galactose (G) on antenna; [3]G1 and [6]G1 indicates that the galactose is on the antenna of the α1–3 or α1–6 mannose; Sx, number (x) of sialic acids linked to galactose.

| Trait | Glycan structure |
| --- | --- |
| P1 | A2G2S2 |
| P2 | A2BG2S2 |
| P3 | FA2G2S2 |
| P4 | FA2BG2S2 |
| P5 | A2[6]G1S1 |
| P6 | A2[3]G1S1 |
| P7 | FA2[6]G1S1 |
| P8 | FA2[3]G1S1 |
| P9 | A2G2[6]S1 |
| P10 | A2G2[3]S1 |
| P11 | A2BG2S1 |
| P12 | FA2G2S1, M5, A2 |
| P13 | FA2BG2S1 |
| P14 | A2B |
| P15 | FA2 |
| P16 | A2[6]G1 |
| P17 | A2[3]G1 |
| P18 | FA2B |
| P19 | A2B[6]G1 |
| P20 | A2B[3]G1 |
| P21 | FA2[6]G1 |
| P22 | FA2[3]G1 |
| P23 | FA2B[6]G1, A2G2 |
| P24 | FA2B[3]G1 |
| P25 | A2BG2 |
| P26 | FA2G2 |
| P27 | FA2BG2 |


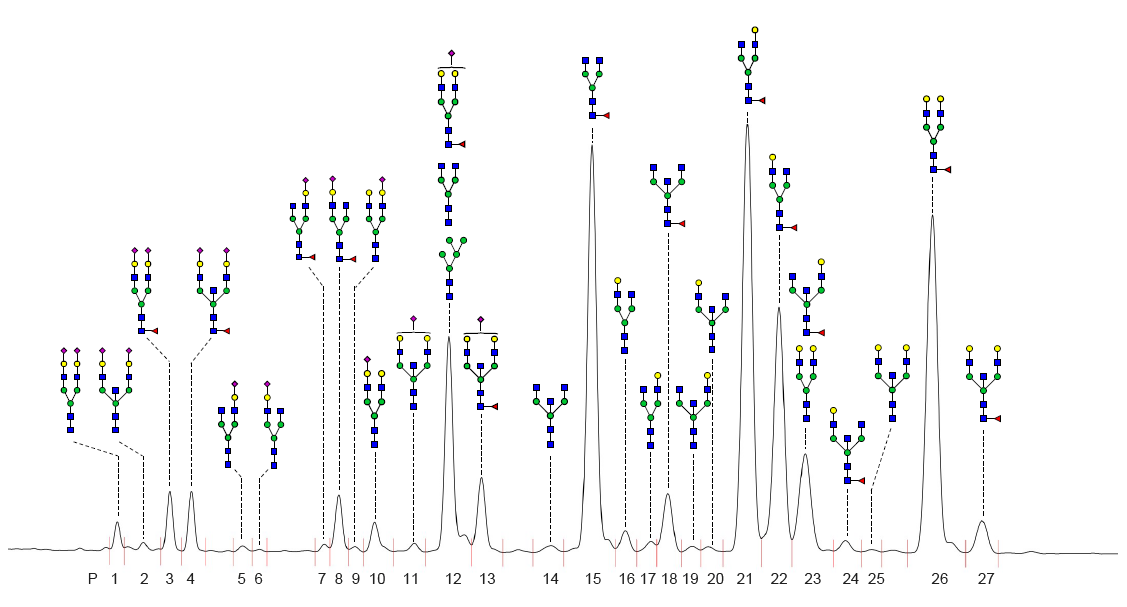


**Supplementary Figure 1. CGE IgG N-glycoprofile – 27 peaks.**

Structural schemes: given according to Consortium for Functional Glycomics (CFG) guidelines in terms of GlcNAc — blue square; mannose — green circle; galactose (β1-4 linked) — yellow circle; N-acetylneuraminic acid — purple diamond, α2,3-linked (directed down left) and α2,6-linked (directed up left); fucose — red triangle.


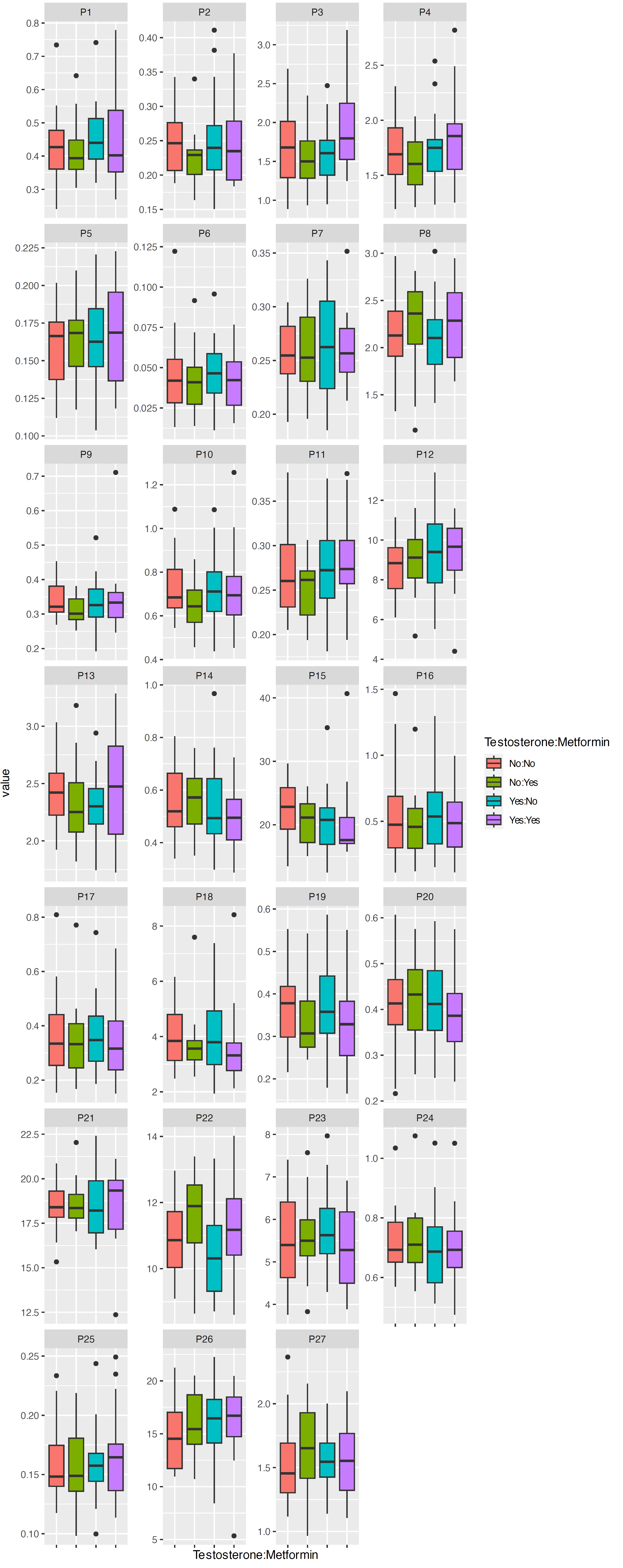


**Supplementary Figure 2. Distribution of 27 N-glycans across the four treatment groups in the clinical trial at the first timepoint.**

Red square – placebo group; green square – group on metformin therapy; blue square – group on testosterone therapy; purple square – group on both testosterone and metformin therapy.
